# *Drosophila* glioma models: a view from the neural stem cell niche

**DOI:** 10.3389/fcell.2026.1854057

**Published:** 2026-07-22

**Authors:** Brooke Kinsela, Olga Zaytseva, Damien Muckle, Teresa T. Bonello, Leonie M. Quinn

**Affiliations:** Genome Science and Cancer Division, The John Curtin School of Medical Research, The Australian National University, Canberra, ACT, Australia

**Keywords:** *Drosophila*, extracellular matrix, glioma, glioma stem cell, neural stem cell, stem cell niche

## Abstract

Gliomas are the most common primary brain tumours in adults, characterised by significant cellular and molecular heterogeneity. Recent advances in molecular profiling have transformed brain tumour classification but, despite these developments, gliomas remain refractory to standard and targeted treatments. The prediction that glioma stem cells (GSCs) drive tumour initiation has focused research efforts on determining intrinsic mechanisms driving acquisition of stemness. Nevertheless, GSCs reside in a cellular environment (or stem cell niche) comprised of differentiated glioma cells, with potential to influence stemness of neighbouring GSCs. Thus, if we are to understand initiation and progression of these stem cell-driven tumours, in addition to elucidating cell intrinsic regulation of GSC fate, we must also delineate extrinsic signalling inputs from the GSC niche. For this, we require *in vivo* models enabling dissection of key lines of communication between GSCs and the surrounding niche. Here we provide an overview of GSC-niche modelling using *Drosophila*, where key mechanisms of neural stem cell control and genetic drivers of glioma are conserved. We focus on the utility of *Drosophila* models for modelling both intrinsic and extrinsic inputs to stem cell fate control, which have provided unique insights into the molecular and cellular basis of glioma initiation and progression.

## 1 Introduction

Gliomas are the most common tumours of the central nervous system (CNS), making up approximately 80% of all primary malignant brain cancers (Louis et al., 2021). Glioma patients typically present with cognitive and neurological dysfunction, including motor defects, fatigue and behavioural changes (IJzerman-Korevaar et al., 2018). Due to infiltrative growth into normal brain tissue, gliomas are particularly challenging to remove by surgery and, therefore, frequently relapse and progress. Moreover, the significant genetic and cellular heterogeneity of these tumours promotes resistance to current treatment regimes. Thus, despite the standard therapy of surgical resection, radiation, and chemotherapy, median survival is approximately 15 months for high-grade glioma patients (Gilbert et al., 2014), while ∼15% of low-grade tumours recur and become more aggressive following therapy (Johnson et al., 2014), likely due to chemotherapy-induced hypermutation (Yu et al., 2021).

**FIGURE 1:**
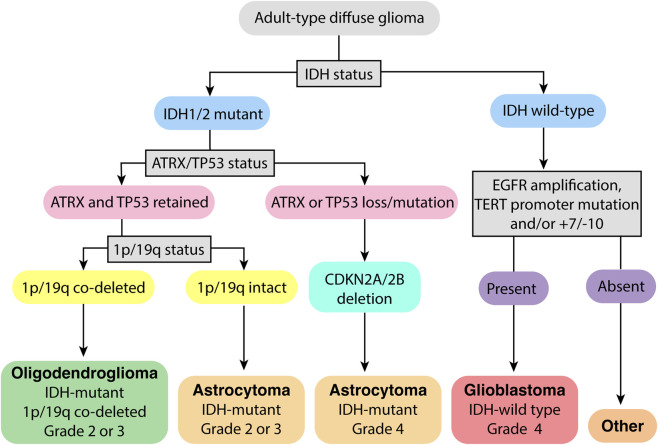
Molecular classification of adult-type glioma. Gliomas carrying a IDH1/2 mutation are further classified based on chromosome 1p/19q status as oligodendroglioma (deleted) or astrocytoma (intact) with mutations in ATRX and TP53 associated with high grade astrocytoma. Deletion of CDKN2A/2B results in grade 4 classification. IDH wild type with additional features including EGFR amplification, TERT promoter mutation and/or chromosome 7 gain/chromosome 10 loss are classified as grade 4 glioblastoma.

Gliomas resemble differentiated glial cells morphologically (e.g., astrocytes or oligodendrocytes) providing the basis to the historical classification. Until recently, histology (including cellular appearance, mitosis, necrosis), was used exclusively to grade glioma aggression, with grade 4 tumours predicted to have the poorest prognosis and grade 1 the best outcomes for patients (Louis et al., 2021). More recently, genome-wide sequencing of glioma tissue, catalogued as part of The Cancer Genome Atlas (TCGA), revealed the extent of molecular heterogeneity within glioma subtypes (Brennan et al., 2013; Network, 2015; Ceccarelli et al., 2016). Moreover, large scale genomic studies uncovered driver mutations that not only inform diagnosis and prognosis but also predict therapeutic response (Olar et al., 2015; Reifenberger et al., 2017; Louis et al., 2021). This revised glioma classification has therefore revolutionised clinical management, with molecular genotyping for biomarker identification routinely used to classify adult gliomas into three major sub-types: oligodendroglioma, astrocytoma and glioblastoma (Louis et al., 2021). For example, isocitrate dehydrogenase (IDH) is wildtype in glioblastoma, IDH1/2 mutations combined with loss of chromosomal arms 1p/19q define oligodendroglioma, while mutant IDH in the absence of 1p/19q is diagnostic for astrocytoma (Figure 1).

Nevertheless, despite improvements in glioma classification, using genomic, transcriptomic, and epigenomic approaches (Brennan et al., 2013; Ceccarelli et al., 2016), we lack functional data linking predicted driver mutations with tumour initiation and/or progression *in vivo*. Moreover, despite the molecular complexity of these tumours, functional evidence for interactions between combinations of predicted driver mutations is even more scarce. To address these gaps in the field, we require genetically flexible models. Here, we discuss how the wide range of genetic tools developed in *Drosophila* can be used to link glioma genotype with phenotype.

## 2 Glioma: tumour mutations hijack developmental programs

Understanding the cellular origin of glioma is vital for elucidating mechanisms underlying the tumour heterogeneity promoting therapeutic resistance and, ultimately, tumour relapse. Early studies revealed that primary brain tumour cells express the cell surface antigen and stem cell marker CD133 and, moreover, have capacity to initiate gliomas in the mouse brain resembling the original patient tumour (Singh et al., 2004; Lathia et al., 2011). Gliomas are, therefore, widely hypothesised to be driven by neural stem cell-like populations, known as glioma stem cells (GSCs) (Persson et al., 2010; Sugiarto et al., 2011; Ceccarelli et al., 2016), which share markers with neural stem cells (NSCs) and can similarly generate neurospheres (Ignatova et al., 2002; Galli et al., 2004; Singh et al., 2004).

The GSC hypothesis is further supported by single cell RNA sequencing (scRNA-seq) of glioblastoma, which identified neural stem and progenitor (NSPC)-like, oligodendrocyte-progenitor (OPC)-like and astrocyte-like signatures (Neftel et al., 2019). Moreover, while oligodendrogliomas histologically resemble differentiated glia (Wesseling et al., 2015), scRNA-seq revealed that, in addition to a predominant mature glial-like signature (oligodendrocytes and astrocytes), a small proportion (∼10%) of oligodendroglioma cells express stem/progenitor signatures (Tirosh et al., 2016). Indeed, in IDH mutant glioma, differentiated glial-like cells are derived from relatively few asymmetrically dividing stem/progenitor-like cells, and differences between astrocytoma and oligodendroglioma are attributed to divergent driver mutations and tumour microenvironment contributions (Venteicher et al., 2017).

Signalling pathways controlling NSC fate during development, such as Notch, Wnt, Shh (Sonic hedgehog), and EGFR (Epithelial growth factor receptor) pathways, are activated in glioma (Purow et al., 2005; Li et al., 2009; Rheinbay et al., 2013; Abiria et al., 2014). Identifying how these networks promote stem/progenitor states will be essential for understanding tumour initiation and progression and may also reveal potential therapeutic targets. To make progress however, we must first understand normal brain development, including interactions between neural stem cells (NSCs) and their surrounding cellular microenvironment, or niche. For this, we require multicellular model systems amenable to genetic manipulation.

## 3 Mammalian NSC lineage models for glioma initiation

**FIGURE 2:**
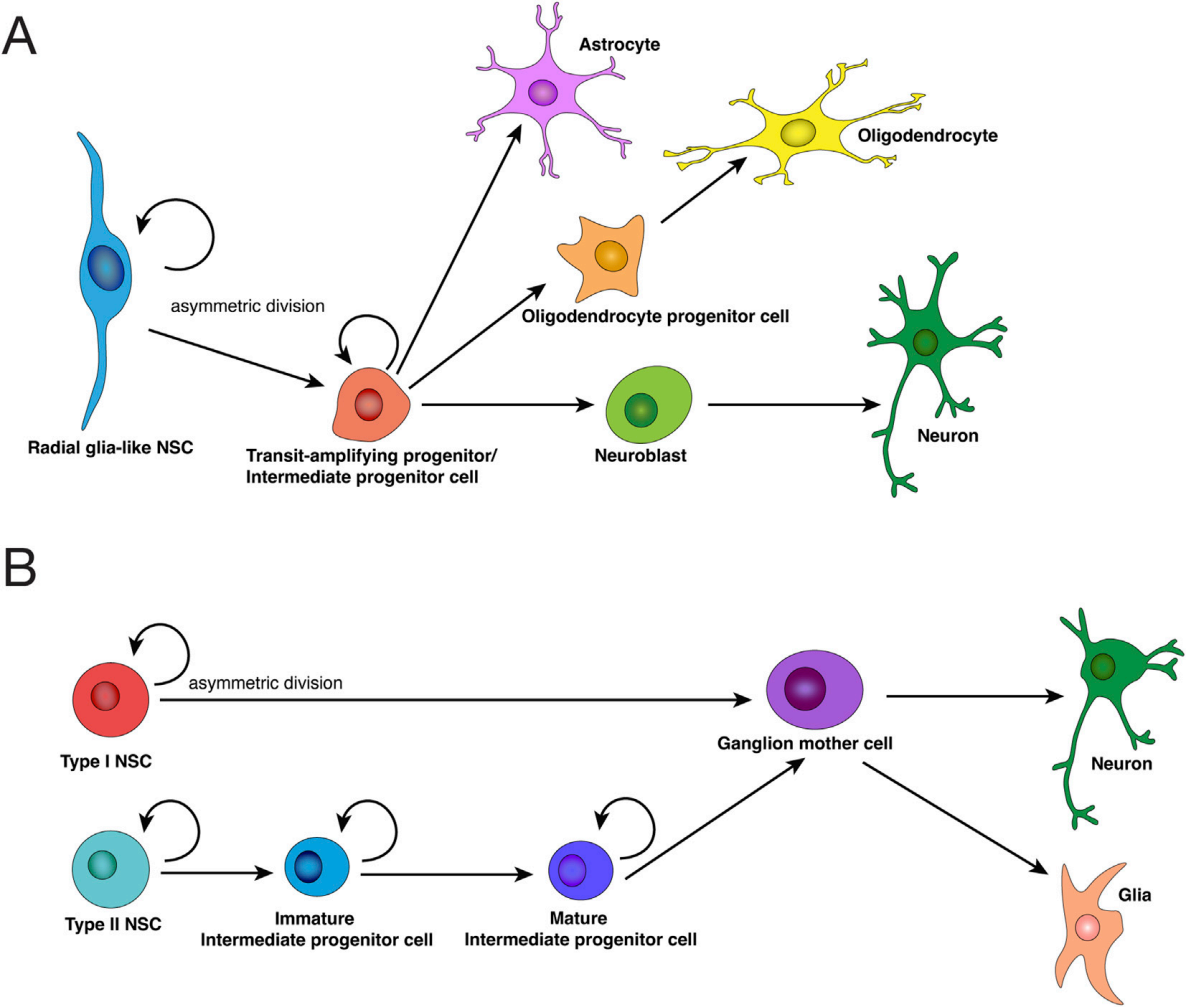
Mammalian and *Drosophila* NSC lineages. **(A)** Radial glia-like NSCs give rise to transit-amplifying progenitors which further differentiate into astrocytes or oligodendrocytes or neurons via lineage-committed progenitors. **(B)** *Drosophila* type I NSCs give rise to ganglion mother cells, while the Type II lineage includes transit-amplifying progenitors that mature and give rise to ganglion mother cells before differentiating into neurons or glia.

In the developing mammalian brain NSCs, known as radial glia, give rise to the central nervous system (CNS) (Gage and Temple, 2013). Symmetric divisions expand the NSC population, while asymmetric divisions generate progenitors with self-renewing capacity capable of differentiating into neurons and glia (astrocytes and oligodendrocytes, Figure 2A) (Mira and Morante, 2020; Gimeno and Paridaen, 2022). The delicate balance between NSC proliferation, differentiation and quiescence is controlled by a combination of cell-intrinsic and cell-extrinsic programs (Renault et al., 2009; Hsieh and Zhao, 2016).

While most neural cells terminally differentiate after the embryonic stage, a small population of stem/progenitor cells remain in the adult mammalian brain (Ming and Song, 2011; Baig et al., 2024). In the CNS, NSCs are localized in the subventricular zone (SVZ) and subgranular zone (SGZ) where they contribute to regeneration of neurons and glia (Doetsch et al., 1999; Gage, 2000). Oligodendrocyte progenitor cells (OPCs) are found throughout the brain and retain their proliferative, migratory, and differentiation potential (Raff et al., 1983; Reynolds and Hardy, 1997; Dimou et al., 2008; Dimou and Gallo, 2015). Although adult human astrocytes are predominantly post-mitotic (Zhang et al., 2016), single cell sequencing analysis identified a small sub-population of proliferative astrocytes residing in the subgranular zone of the hippocampus (Batiuk et al., 2020). Moreover, adult astrocytes retain limited capacity to proliferate in the context of brain injuries (Ge et al., 2012; Bardehle et al., 2013). Given their proliferative capacity, both NSCs and lineage-committed progenitors have been proposed to directly drive glioma initiation (Persson et al., 2010; Sugiarto et al., 2011; Ceccarelli et al., 2016).

Indeed, genetic mouse models support neural stem and/or progenitor cell (NSPC)-like origins for glioma. For instance, glioblastoma driver mutations such as p*TERT* (*TERT* promoter), *p53*, *Pten* and *Egfr*, enable migration of adult NSCs outside the subventricular zone to result in high-grade gliomas in distant brain regions in murine models (Lee et al., 2018). Furthermore, *p53* and *Pten* depletion from the subventricular zone of the mouse brain results in tumours positive for OPC markers, such as OLIG2 and PDGFRa, and negative for markers of mature neural lineage cells (e.g., NeuN for neurons, MBP for oligodendrocytes, and GFAP for astrocytes) (Lee et al., 2018).

In support of OPC glioma origin, concurrent mutations in the tumour suppressors *p53* and *NF1* in NSCs transform daughter OPC progenitors into tissue resembling malignant glioma (Liu et al., 2011). Likewise, loss of *p53* and *Pten* tumour suppressors in Olig1/2+ intermediate progenitors results in invasive high-grade gliomas (Verma et al., 2023). In accordance with this, Olig2, a transcription factor and fate-determinant in OPCs, is highly expressed in low grade glioma and is essential for promoting glioma in murine models (Ligon et al., 2004; Ligon et al., 2007; Tian et al., 2025). In human NSCs, CRISPR targeting of *TP53*, *NF1* and *PTEN* tumour suppressors increased expression of stem/progenitor markers typically elevated in glioma (e.g., hNES and OLIG2) and generate high-grade glioma capable of invading the subventricular zone and white matter in the mouse brain (Wang et al., 2021). Thus, glioma mutations have capacity to drive high-grade tumours via impaired differentiation of stem and/or progenitor cells.

## 4 *Drosophila* NSC models for GSC-driven gliomagenesis

The flexible genetics, and conservation of developmental signalling pathways controlling NSC fate (e.g., Notch, Wnt) (Geissler and Zach, 2012; Mirzoyan et al., 2019), has enabled glioma modelling using *Drosophila* NSCs (also referred to as neuroblasts). In the developing larval brain, NSCs are classified as type I or type II (Figure 2B). Type I NSCs divide asymmetrically to self-renew and produce a daughter ganglion mother cell (GMC), which terminally differentiates into neurons and/or glial cells (Homem and Knoblich, 2012). Like mammalian radial glial lineages (Figure 2A), type II NSCs generate neurons and/or glia via transit amplifying intermediate neural progenitor cells (INP) (Kang and Reichert, 2015). Thus, asymmetric division of NSCs maintains the stem cell pool and, concurrently, enables differentiation into neurons and glia, populating the central nervous system for brain development (Knoblich, 2008; Li et al., 2014; Harding and White, 2018).

As defective NSC renewal or differentiation can lead to stem cell loss or overproliferation, asymmetric division of NSCs is tightly regulated (Caussinus and Gonzalez, 2005; Bowman et al., 2008; Chia et al., 2008; Knoblich, 2010). Forward genetic screens provided an unbiased approach for discovery of pathways regulating neural stem and progenitor cell (NSPC) fate in *Drosophila*. For example, tumour suppressor mutations, such as brain tumor (*brat*) and prospero (*pros*), were identified by screening for brain tumour phenotypes associated with NSPC differentiation defects (Caussinus and Gonzalez, 2005; Betschinger et al., 2006; Bowman et al., 2008). Moreover, *brat* knockdown specifically in NSCs is sufficient to drive tumorigenesis in the adult brain (Mukherjee et al., 2016). Brat normally prevents brain tumours by functioning as a post transcriptional inhibitor of the *MYC* oncogene homologue, dMyc (Betschinger et al., 2006), and by repressing Notch pathway activity (Mukherjee et al., 2016). In mammals, Brat is conserved as tripartite motif-containing (TRIM) protein family members TRIM3 and TRIM32 (Giannopoulou et al., 2022), which are required for neural progenitor differentiation in the mouse brain and, moreover, frequently mutated in human glioma (Schwamborn et al., 2009; Mukherjee et al., 2016). Like Brat, TRIM3 functions as a tumour suppressor in glioma cells by controlling the *MYC* oncogene and activity of the Notch signalling pathway (Chen et al., 2014; Mukherjee et al., 2016).

Forward genetic screens in *Drosophila* also identified loss-of-function mutations in tumour suppressor gene *lethal giant larvae* (*lgl*) as drivers of brain neoplasms (Gateff, 1978; Bilder et al., 2000). In NSCs, lgl protein is phosphorylated by apical aPKC (atypical protein kinase C), which restricts lgl function to the basolateral membrane, enabling recruitment of cell fate determinants (e.g., miranda) for asymmetric division (Ohshiro et al., 2000; Peng et al., 2000; Albertson and Doe, 2003; Betschinger et al., 2003). Thus, *lgl* mutant NSCs undergo symmetric division (Lee et al., 2006), while aPKC activation drives expansion of neural progenitors (Paglia et al., 2017), with either resulting in brain tumour phenotypes. Lgl’s tumour suppressor function is conserved between *Drosophila* and mammals, with human LGL1 phosphorylated and inactivated in glioblastoma, while constitutively active LGL1 induces differentiation of GSCs (Gont et al., 2014). Thus, NSC fate control mechanisms, uncovered using forward genetic screens in *Drosophila*, are conserved, and likely required to regulate GSC renewal and prevent glioma.

## 5 Reverse genetics studies in *Drosophila* recapitulate high-grade glioma

Glioblastoma, the most lethal glioma subtype (Louis et al., 2021), has been modelled using reverse genetic approaches in *Drosophila*. For example, the EGFR Receptor Tyrosine Kinase pathway is amplified in ∼45% of glioblastoma, notably by the hyperactivating truncating mutant form, EGFRvIII, which is found in ∼50% of all EGFR-amplified glioblastomas (Brennan et al., 2013). The outcome is ligand-independent constitutive signalling and activation of downstream PI3K/Akt and Ras/MAPK pathways (Nishikawa et al., 1994; Huang et al., 2001; Brennan et al., 2013). Alternate pathways for glioma progression via RTK activation include loss of PI3K-Akt inhibitor, PTEN (Groszer et al., 2001; Parsons et al., 2008; Network, 2015) or activation of the PI3K catalytic subunit, encoded by *PIK3CA* (Samuels et al., 2004; Network, 2015).

To model EGFR/PI3K-driven high-grade glioma, the pan-glial *repo*-GAL4 (reversed polarity) driver was used to express activated EGFR and PI3K transgenes, which results in glial lineage overproliferation and disorganisation reminiscent of neoplastic glioblastoma phenotypes (Read et al., 2009; Witte et al., 2009). Co-activation of RTK homologs of human PDGFR/VEGFR and FGFR1 (pvr [PDGF- and VEGF-receptor related] and htl [heartless], respectively) with *repo*-GAL4 also results in brain tumour phenotypes (Witte et al., 2009). Consistent with inactivation of the retinoblastoma (RB) tumour suppressor in glioblastoma (Classon and Harlow, 2002; Chkheidze et al., 2021), RTK-driven brain tumours are enhanced by co-knockdown of the RB homologue, Retinoblastoma family protein 1 (Rbf1) (Stevaux et al., 2002; Read et al., 2009). Moreover, expression of Raf in *Drosophila* glial cells has served as a model of low-grade glioma, driving relatively moderate increases to total glial cell number, mitotic index and brain size (Penman et al., 2015; Chen et al., 2018).

Despite high prevalence of EGFR and/or PI3K mutations in glioma, targeting with inhibitors has, thus far, not improved patient outcomes (Pearson and Regad, 2017). As targeting downstream pathways might provide an alternate therapeutic strategy, kinome-wide screens were performed to identify modifiers of the EGFR/PI3K brain tumour phenotype, with enhancers including serine/threonine kinases Hippo and Warts that function to inactivate the downstream transcriptional co-activator Yorkie (Yki) (Read et al., 2013). Consistent with this, Yki expression is increased in the EGFR/PI3K glioma model and is, moreover, required to drive tumorigenesis (Vigneswaran et al., 2021). These observations translate to EGFR-activated glioblastoma, where targeting of the Yki homologue YAP and the related transcriptional co-activator TAZ with Verterporfin has been shown to slow tumour progression and extends median survival in mouse xenograft models (Vigneswaran et al., 2021). Thus, pathways identified downstream of EGFR-activation in *Drosophila* models are also required for glioma progression in mammalian systems and have potential to inform alternate avenues for targeting EGFR-positive tumours in patients.

## 6 Neurogenic niches control stem cell fate: from normal development to glioma

The pan-glial models (described above) have shed light on mechanisms driving gliomagenesis but, as glia can also form a cellular niche for NSCs, aberrant glial function also has potential to drive glioma progression cell extrinsically, i.e., by disrupting NSC-niche communication. Indeed, in the context of glioma, GSCs can promote malignant progression in response to neurotrophic factor signalling (Wang et al., 2018) and microenvironment remodelling (Uneda et al., 2021). Thus, in addition to cell intrinsic mechanisms controlling GSC fate, extrinsic cues and support from the surrounding cellular environment will be critical for controlling gliomagenesis (Filatova et al., 2013; Akindona et al., 2022).

The concept of a stem cell niche was first proposed in 1978 (Schofield, 1978) and early evidence for the importance of the niche provided by studies in *Caenorhabditis elegans*, where removal of germline stem cells from their niche results in loss of stemness and aberrant differentiation (Kimble and White, 1981). Subsequent studies in *Drosophila* ovarian germline stem cell models revealed molecular mechanisms of niche-dependent stem cell fate regulation, demonstrating that cell-cell contact (E-cadherin) and secreted cellular signals (Dpp [Decapentaplegic]/BMP [Bone morphogenetic protein]) from the niche, control stem cell renewal and differentiation (Xie and Spradling, 1998; Xie and Spradling, 2000; Song and Xie, 2002). These studies provided the first *in vivo* demonstration that cell-cell contact (Xie and Spradling, 2000) and signalling (Xie and Spradling, 1998) from the niche are both essential for control of asymmetric stem cell division, with defective communication resulting in stem cell loss or, conversely, tumour phenotypes (Losick et al., 2011).

**FIGURE 3:**
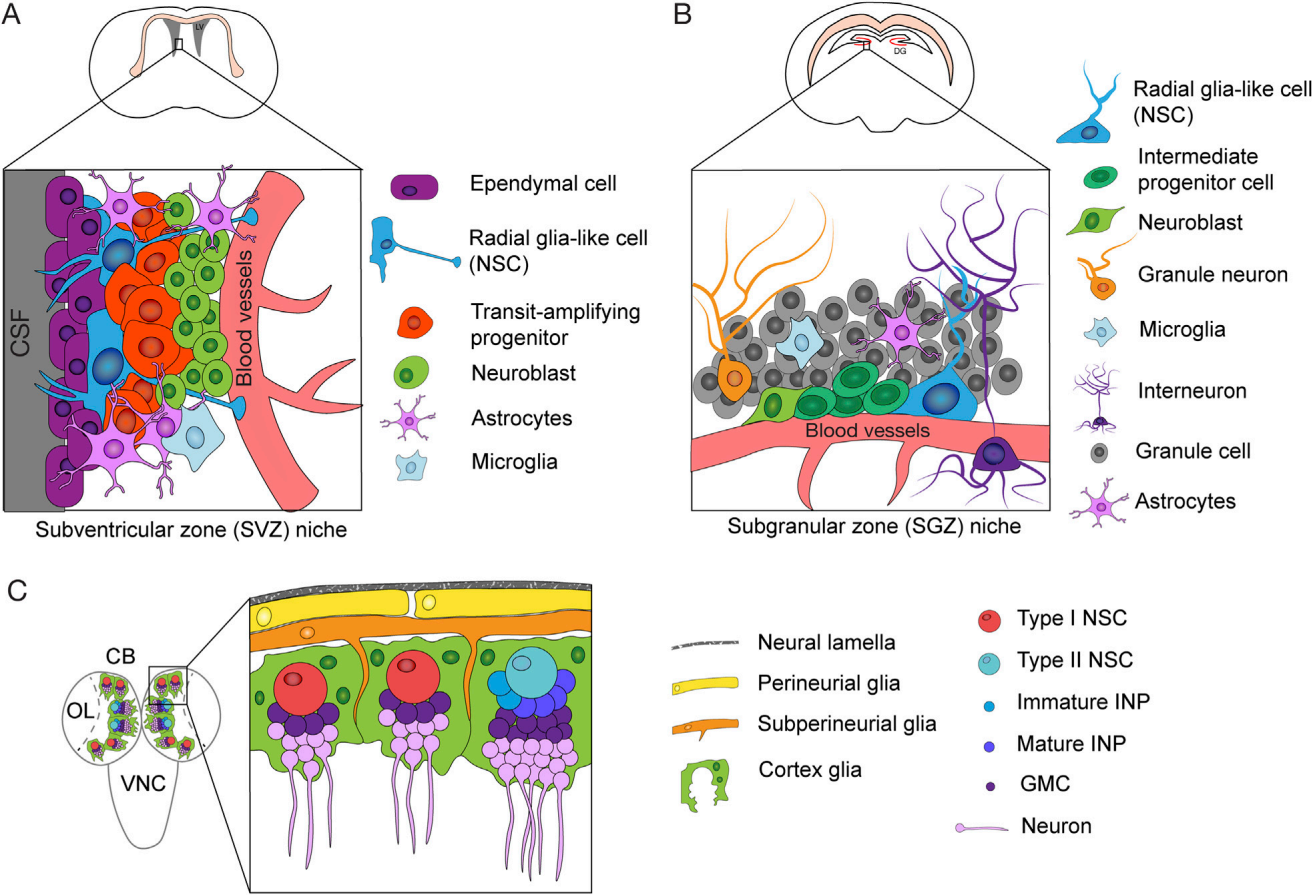
Specialised niches support NSCs in mammals and *Drosophila*. **(A)** Subventricular zone in the adult mammalian brain, where radial glia-like NSCs and their progeny (transit-amplifying progenitors and neuroblasts) are supported by ependymal cells, astrocytes and microglia. **(B)** Subgranular niche in the mammalian hippocampal dentate gyrus supports generation of granule cells from radial glia-like NSCs. **(C)** *Drosophila* larval NSCs, supported by cortex glia, perineurial glia and subperineurial glia, give rise to intermediate progenitor cells (INP) and ganglion mother cells (GMC) which differentiate into neurons and glia.

In the adult mammalian brain, NSCs reside in a complex niche comprised of glial cells (astrocytes and oligodendrocytes), resident immune cells (microglia and macrophages), vasculature, neurons, and extracellular matrix (ECM) components (Llorente et al., 2022). Adult NSCs are supported by two main neurogenic niches (Figure 3): the subventricular zone (SVZ) of the lateral ventricle and the subgranular zone (SGZ) of the hippocampal dentate gyrus (Alvarez-Buylla and Lim, 2004). The SVZ niche supports radial glia-like NSCs, which contact blood vessels and cerebrospinal fluid via basal and apical projections, respectively (Mirzadeh et al., 2008). The SVZ type NSCs generate transit-amplifying progenitors that, after several rounds of proliferation, mature into neuroblasts that migrate prior to further differentiation into neurons and glia (Figure 3A) (Doetsch et al., 1999; Mirzadeh et al., 2008; Wang et al., 2011). Radial glia-like NSCs are also found in the SGZ niche, where they give rise to intermediate progenitor cells that undergo multiple divisions prior to differentiating into neuroblasts (Figure 3B) (Seri et al., 2001), which migrate and mature into excitatory neurons (Cameron et al., 1993; Praag et al., 2002; Abbott and Nigussie, 2020) or mature glia, including astrocytes and oligodendrocytes (Artegiani et al., 2017).

GSCs reside in tumours predominantly comprised of differentiated glial-like cells (Canoll and Goldman, 2008; Tirosh et al., 2016) and experiments using microfluidic chip models, aimed to recapitulate GSC-niche interactions, show GSCs associated with niche astrocytes are more tumorigenic compared with GSCs lacking a glial niche (Adjei-Sowah et al., 2022). To understand GSC-driven tumours we must, however, first delineate interactions between NSCs and glia during normal development. In the adult mammalian CNS, signalling from surrounding glia has been implicated in control of NSC fate. *In vitro* NSC-renewal and proliferation assays, using co-culture with niche astrocytes, indicate that olfactory bulb glia restrict, while SVZ-glia promote, renewal of murine NSCs, with expression profiling identifying Wnt7a as a key factor secreted by SVZ astrocytes promoting NSC renewal *in vitro* (Moreno-Estellés et al., 2012). BMP signalling maintains adult NSC niches in both the SVZ and SGZ (Bond et al., 2012), with BMP4 ligands secreted from astrocytes capable of driving NSCs toward a glial cell fate in the adult CNS (Lim et al., 2000; Peretto et al., 2004; Cole et al., 2016). NSC-glial niche signalling will, therefore, be critical for NSC fate control during normal brain development, while tumorigenic niches are likely to promote glioma initiation, progression, and relapse.

## 7 *Drosophila* glial niche-NSC interactions inform glioma initiation

The simpler NSC niche in *Drosophila*, predominantly comprised of glia (Figure 3C), provides a genetically pliable system for determining how NSC fate is shaped by glial interactions *in vivo*. The niche comprises ensheathing glia, surface glia (perineurial and subperineurial), astrocyte-like glia, and cortex glia (Figure 3C). The outermost layer of the blood-brain barrier comprises a discontinuous sheet of perineurial glia, which deposit a lamella to protect the CNS, and interface with the haemolymph to facilitate uptake of nutrients and removal of dead cells (Volkenhoff et al., 2015; Hunter et al., 2020). This is analogous with endothelial cell function in humans, which form tight junctions and, thus, a barrier preventing the passive diffusion of molecules into the brain (Alahmari, 2021). Ensheathing and astrocyte-like glia interact with neuronal axons and cell bodies to define neural tracts (Freeman, 2015; Yildirim et al., 2019; Corty and Coutinho-Budd, 2023; Valamparamban and Spéder, 2023). Cortex glia directly interact with NSCs and, together with their surface glial progeny (perineurial and subperineurial), provide the predominant component of NSC niche (Figure 3C).

The subperineurial glia form a sheath enveloping the CNS, with septate junctions at cell boundaries controlling trafficking of molecules to the CNS (Stork et al., 2008; Schwabe et al., 2017). By extending long processes into the central brain, between the cortex glial chambers that encase NSCs, subperineurial glia physically contact the neurogenic niche (Contreras et al., 2024). Through this communication with the cortex glial network, subperineurial glia transfer nutrients controlling NSC fate (Dumstrei et al., 2003; Kanai et al., 2018). For example, subperineurial glia secrete Insulin-like peptides (dIlps), in response to nutrition-dependent signalling from the fat body, to stimulate PI3K/Akt signalling and promote NSC reactivation from quiescence during larval development (Chell and Brand, 2010; Sousa-Nunes et al., 2011; Spéder and Brand, 2014; Yuan et al., 2020). Insulin-like growth factor-1 (IGF-1) and IGF-1 receptor (IGF-1R) also promote NSC proliferation in mammals (Arsenijevic et al., 2001). In glioblastoma, increased insulin-like growth factor signalling is implicated in proliferation, invasion, and chemotherapy resistance (Oliva et al., 2018; Nakhaei et al., 2026).

Cortex glia form a critical part of the neurogenic niche, developing membrane projections that, through expansion and remodelling, encapsulates the NSC lineage during late larval development (Pereanu et al., 2005; Spéder and Brand, 2018; Rujano et al., 2022). Cell surface cortex glia proteins, wrapper and Neurexin-IV, connect with the NSC lineage to control cell adhesion and niche architecture (Banach-Latapy et al., 2023). In oligodendroglioma, decreased Neurexin-IV ortholog, CNTNAP2, is associated with tumour recurrence and poor survival (Rautajoki et al., 2022).

Of note, given PI3K activity drives glioma progression (Network, 2008; McBride et al., 2009), nutrition-dependent dIlp secretion from adjacent subperineurial glia activates PI3K/Akt signalling to drives cortex glial growth and membrane expansion (Spéder and Brand, 2018). This communication is bidirectional, with PI3K/Akt promoting NSC reactivation and expansion, while also driving cortex glial membrane expansion to encase the expanding NSC lineage (Spéder and Brand, 2018; Yuan et al., 2020). The microcephaly protein wdr62 (WD-repeat domain 62) is essential in larval glia for maintaining NSCs, promoting brain growth and development (Lim et al., 2017) in a manner dependent on Akt/PI3K signalling (Shohayeb et al., 2020). In 2D culture and mouse xenograft models of glioblastoma, increased exosomal circular RNA encoding WDR62 correlates with temozolomide resistance and disease progression (Geng et al., 2022).

*Drosophila* studies provide insight into roles of conserved pathways, including Hedgehog, PI3K, and Wnt, in gliomagenesis (Takezaki et al., 2011; Bazzoni and Bentivegna, 2019). Wg (wingless) secreted by surface (perineurial and subperineurial) glial cells, activates canonical Wg/Wnt signalling in NSCs to promote exit from quiescence and enable brain development (Lin et al., 2025). Alk (Anaplastic lymphoma kinase) maintains PI3K signalling in NSCs via the ligand, Jelly belly (Jeb), which is expressed in glia and required for NSC growth (Cheng et al., 2011). In association with lipid metabolism, glial-derived hedgehog (Hh) signalling regulates NSC fate, with Hh knockdown in glia reducing proliferation (Dong et al., 2021; Sood et al., 2024). In glioblastoma the Hh/Gli1 (GLI family zinc finger 1) signalling pathway inhibitor drug, vismodegib, induces apoptosis and cell cycle arrest in glioblastoma neurospheres *in vitro* (Chandra et al., 2015).

Netrins are also secreted by cortex glia, and function cell non-autonomously to modulate NSC fate via Robo1 kinase and the small GTPases, Rac1 and Cdc42. As Cdc42 is a physical partner of Par-6, a core component of the Par (Par-6-aPKC-Par3/Bazooka) apical complex in mitotic NSCs, defective Netrin/Robo1 pathway function impairs asymmetric NSC division (Torres-Jurado et al., 2022). In addition to these paracrine signalling networks, the cortex glial niche provides NSCs with environmental protection from nutrient restriction and oxidative stress (Cheng et al., 2011; Bailey et al., 2015).

In addition to the critical function of glial cells in the regulation of NSC fate, communication from the niche is required for maintenance of mature neurons in the context of glioma. The growth of malignant gliomas is often associated with progressive neurological dysfunction (Krishna et al., 2023). In human astrocytoma, ultra-long cellular protrusions, or tumour microtubes, meditate cell-cell communication (Osswald et al., 2015). *Drosophila* glioma models i.e., co-overexpression of constitutively active EGFR and PI3K driven with pan-glial repo-GAL4 [see Section 5 (Read et al., 2009; Witte et al., 2009)], similarly develop networks of actin-rich membrane projections, referred to as tumour microtubes (TMs) that surround neurons to isolate neural clusters into discrete “nests” (Portela et al., 2019). The membrane contact between these glial microtube (TM) networks sequester, or “vampirize,” Wg away from neurons. Moreover, Wg pathway activation in TMs results in a positive feedback loop, whereby Jun N-terminal kinase (JNK) upregulates matrix metalloproteinases (MMPs), to drive further neural degeneration via TM expansion and infiltration into normal brain tissues (Portela et al., 2019; Portela et al., 2020). *Drosophila* models, therefore, shed light on glial lineage networks controlling NSC and mature cell fate *in vivo*. The conservation of these networks in mammalian systems, and dysregulation in glioma, suggests these pathways will be critical for cell non-autonomous control of GSC fate by glial-like cells in glioma.

## 8 Emerging roles for the extracellular matrix (ECM) in NSC fate control

By bridging the space between the niche cells and NSCs, the extracellular matrix (ECM) provides both physical support and function as a reservoir for secreted signalling factors essential for controlling NSC fate and brain development. The matrisome encompasses core architectural proteins (e.g., collagens, glycoproteins and proteoglycans) and factors required for ECM synthesis and remodelling (Naba et al., 2012). The ECM is highly specialised across different tissue and organ microenvironments. In contrast with other organs, fibrous components (e.g., collagens and fibronectins) are highly underrepresented in the human brain, where proteoglycans, glycoproteins and glycosaminoglycans (GAGs) constitute the bulk of the neural ECM (Tewari et al., 2022). As the ECM is critical for cell-cell communication and local microenvironment organisation, dysregulation is expected to promote gliomagenesis.

The ECM is essential for propagating and integrating cell-cell communication signals required for nervous system development. Unique ECM structures (perineuronal nets) function in the adult mammalian brain parenchyma, the region outside of the NSC niche housing differentiated neural cells, to restrict neuronal plasticity (Wang and Fawcett, 2012). High-resolution proteomic analysis revealed differences between the ECM of the NSC niche and parenchyma, including matrisome signatures for the non-neurogenic somatosensory cortex, the olfactory bulb, and the SVZ, where most NSCs reside (Kjell et al., 2020). Matrisome-associated proteins were enriched in the SVZ and, somewhat surprisingly, quiescent NSCs, but not activated NSCs, transit-amplifying progenitors or neuroblasts, were the predominant source of local ECM production (Kjell et al., 2020).

The core mammalian matrisome genes are conserved in *Drosophila,* including three collagens, 21 glycoproteins and three proteoglycans, as well as 219 matrisome-associated genes (Davis et al., 2019). Much of our understanding of ECM patterning and function in the fly nervous system has emerged from studies of barrier formation, which is mediated by specialised glial-ECM interactions (Fernandes et al., 2024). Among these, the neural lamella, a dense network of ECM components that interacts with surface glia to sustain integrity of the blood-brain barrier, are the most well characterised.

The proteoglycan family regulates neural progenitor proliferation (Long and Huttner, 2019). Mouse embryos lacking perlecan, a heparan sulfate proteoglycan (HSPG), develop microcephaly due to reduced progenitor proliferation (Girós et al., 2007), in line with an earlier observation in *Drosophila*, where mutations in the perlecan homologue, trol (terribly reduced optic lobes), reduces NSC proliferation (Park et al., 2003). In both studies, consistent with the role of HSPGs as co-receptors for growth factor signalling, perlecan/trol establish concentration gradients for sonic hedgehog/hedgehog ligands required for NSPC renewal (Park et al., 2003; Girós et al., 2007). Perlecan is also increased in glioblastoma and correlates with poor patient outcomes (Kazanskaya et al., 2018). *Drosophila* HSPG, Dally-like (Dlp), is expressed in surface glia where it activates Gbb/BMP signalling, to promote NSC proliferation and brain growth (Kanai et al., 2018). Similarly, loss of glypican-1 in mice reduces brain size, a consequence of early defects in neurogenesis (Jen et al., 2009). Recent high-resolution characterisation of ECM composition in adult and paediatric high-grade glioma highlights the prominent representation of proteoglycan family members within the ECM landscape (Day et al., 2025).

ECM glycoproteins are also required for *Drosophila* neurogenesis. For example, secretion of the glycoprotein anachronism (ana) from glia maintains postembryonic neuroblasts in a quiescent state (Ebens et al., 1993), while Drospondin (*Drosophila* Spondin 1 homologue) is expressed in glia and required for mushroom body development (Rojo-Cortés et al., 2026) and Spondin1 is overexpressed in glioma (Towner et al., 2013). Furthermore, activation of quiescent neural progenitors by acute injury in the adult fly brain, is associated with upregulation of the glycoprotein Swim (Simões et al., 2022). Swim binds to, and increases solubility of, lipid-modified Wg/Wnt *in vitro*, to drive neural progenitor expansion (Mulligan et al., 2012; Simões et al., 2022). Thus, understanding how glycoproteins control normal development and nervous system repair, will shed light on mechanisms of NSC regulation by the ECM and dysregulation in context of glioma.

## 9 ECM dysregulation promotes glioma invasion

Glioma aggression and therapeutic resistance is enabled by extensive infiltration into the adjacent brain parenchyma. Delineating invasive front interactions with the ECM will be essential for understanding glioma spread and determining how residual niches, persisting after tumour debulking surgery, might contribute to therapeutic resistance. Models of collective cell migration during *Drosophila* development have enabled identification of pathways controlling glioma invasion. For example, conserved regulators of GSC invasion were predicted by intersecting genes associated with decreased survival in GBM patients with those required for border cell migration (a cohesive cluster of 6–10 epithelial follicle cells). Importantly, functional analysis revealed the conserved small GTPases, Cdc42 and Rap1a, are required for GSC migration (Volovetz et al., 2020).

Mathematical modelling at the invasive tumour front in the *Drosophila* EGFR/PI3K glioma models revealed integrin activation and accumulation of the matrix metalloprotease MMP1 at the leading edge (Conte et al., 2021). MMPs, zinc-dependent enzymes crucial for breaking down components of the ECM, like collagen and elastin, are also secreted by astrocytes, being required for normal function and dysregulated in disease (Kinoshita et al., 2013; Reinhard et al., 2015). For example, overexpression of MMP-1 in adult mice disrupts the balance between neural stem and progenitor cells, increasing hippocampal NSCs while reducing oligodendrocyte progenitors (Valente et al., 2015).

## 10 Altered mechanical properties of the ECM drives gliomagenesis

Altered tissue mechanics are a fundamental feature of all solid tumours, including gliomas. Increased stiffness of tumours, relative to the surrounding normal tissue, can occur because of overproliferation within a confined space. The stiffness and associated changes in ECM composition can further rewire signalling programs controlling proliferation, survival, invasion, and therapy resistance. Progression from low- to high-grade glioma is associated with an increase in tissue stiffness, ranging from 100 to 10^4^ Pa (Miroshnikova et al., 2016), which is recapitulated by *Drosophila* low grade- and high grade-like glioma models. Consistent with increased collagen deposition and active FAK signalling, atomic force microscopy revealed the lowest stiffness for non-transformed larval brains, while glial-specific expression of activated Raf, the human FGFR3-TACC3 fusion gene, and constitutively active EGFR and PI3K, progressively increased stiffness in order of tumour severity (Chen et al., 2018).

Integrins, heterodimeric transmembrane protein complexes, enable tumour cells to sense, and transduce, mechanical forces by interacting with ECM proteins. Intracellularly, integrins recruit adaptor proteins and kinases of the focal adhesion complex, creating a mechanical link to the actin cytoskeleton, converting mechanical force into biochemical signals (Puklin-Faucher and Sheetz, 2008; Sun et al., 2016). Mechanical forces can activate transcriptional programs to modulate cell proliferation and differentiation. For example, the YAP and TAZ transcriptional regulators respond to mechanical inputs, including membrane forces, cell density or cell stretch, shear stress and ECM rigidity (Panciera et al., 2017). In mammals, YAP/TAZ regulate progenitor proliferation and astrocyte differentiation downstream of integrin and integrin-like kinase (Chen et al., 2024). In *Drosophila*, overexpression of the Yki ortholog of YAP/TAZ in NSCs increases proliferation and brain overgrowth (Poon et al., 2016). Furthermore, brain overgrowth in the high-grade *Drosophila* glioma model, where coactivation of Ras/PI3K drives NSC expansion, is suppressed by Yki knockdown (Gangwani et al., 2020), suggesting mechanical inputs will control glioma progression.

Mechanical tensions are also sensed by ion channels at the cell membrane. For example, evolutionary conserved Piezo channels function as primary mechanosensors, undergoing conformation changes to permit cation influx, thereby converting changes in membrane tension into intracellular signals. In *Drosophila*, while Piezo transmembrane channels are dispensable for normal glial cell proliferation, glial tumour models depend on Piezo for increased tissue stiffness and tumour growth (Chen et al., 2018). The relevance of these observations in flies, is highlighted by overexpression of the human ortholog PIEZO1 in high grade glioma, and inverse correlation with patient survival (Chen et al., 2018). Taken together, the interaction between *Drosophila* GSCs and glial niche mechanics provide novel insights into gliomagenesis, warranting future investigation.

## 11 Future directions: expanding models to recapitulate the genetics of low-grade glioma

**FIGURE 4:**
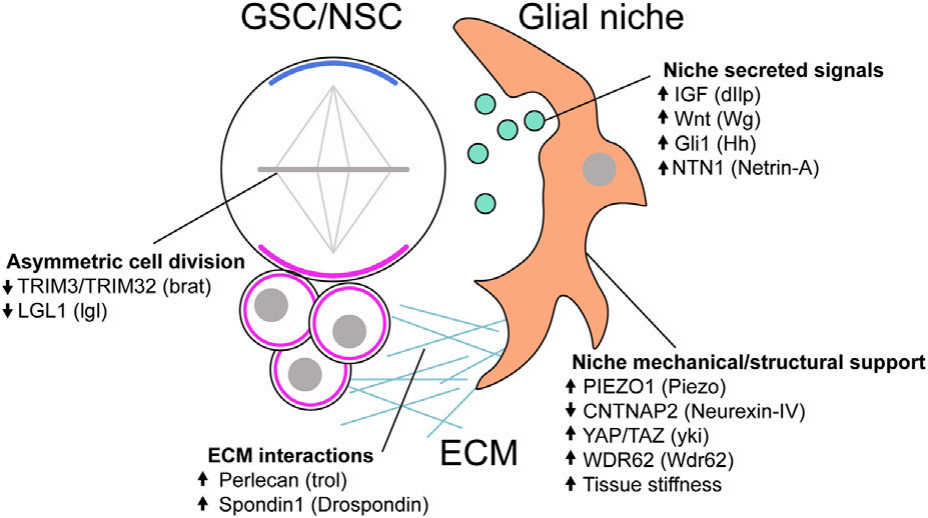
*Drosophila* factors controlling NSC fate conserved in mammals and dysregulated in glioma. Human orthologs and direction of change in glioma and corresponding *Drosophila* ortholog listed in parentheses.

As summarised in Figure 4, *Drosophila* brain models have uncovered new mechanisms of NSC fate control, to provide significant insight into mechanisms of stem cell driven glioma (Zuchero and Barres, 2015; Kanai et al., 2018; Ramon-Cañellas et al., 2018; Spéder and Brand, 2018). However, despite the genetic amenability of *Drosophila*, molecular modelling of low-grade glioma has been limited. In particular, modelling of *IDH* point mutations, drivers of the major classes of low-grade glioma (Wiestler et al., 2013; Louis et al., 2021), have not been reported. Future studies, using the analogous Idh point mutation, in combination with concurrent driver mutations, will enable modelling of the most common low-grade gliomas, oligodendroglioma and astrocytoma. For example, in the background off the Idh mutation, oligodendroglioma can be modelled via co-depletion of conserved transcriptional regulators Capicua (CIC) and Far-upstream binding protein 1 (FUBP1) (Bettegowda et al., 2011), while astrocytomas via mutations in the *TP53* tumour suppressor and chromatin remodelling factor *ATRX* (Burger et al., 2001; Wesseling et al., 2015; Ceccarelli et al., 2016). Modelling of the molecular alterations of IDH1-driven glioma in specific neural lineages will provide insight into mechanisms of low-grade glioma progression.

Glioma treatment has advanced little over the past 3 decades (Aldape et al., 2019), at least in part due to the intricate biomolecular interactions in the brain, integrating developmental, genetic, and microenvironmental cues (Wang et al., 2017; Greenwald et al., 2024). To improve treatment efficacy, we must delineate mechanisms of glioma initiation, progression and relapse, which will include interactions between the GSCs and their cellular niche. With intrinsic regulatory cues and extrinsic structural/signalling support from the niche functionally conserved in *Drosophila* (Egger et al., 2008; Homem and Knoblich, 2012; Corty and Coutinho-Budd, 2023), fly models will continue to serve as a powerful model for dissecting the cellular and molecular basis of gliomagenesis.
